# Allergies in Animals and Humans

**DOI:** 10.3390/vetsci5010005

**Published:** 2018-01-15

**Authors:** Robert Siebers

**Affiliations:** Department of Medicine, Wellington School of Medicine and Health Sciences, University of Otago, 23 Mein Street, Newtown, Wellington 6242, New Zealand; rob.siebers@otago.ac.nz

Allergy to inhalant and food allergens affects many patients worldwide. Various animal species are also known to suffer from allergic diseases, such as dogs and cats with atopic dermatitis. There are many similarities between animals and humans in the pathogenesis, mechanisms, and treatment of allergic diseases. Also research into elucidating allergy mechanisms and development of new therapies in humans has been possible by utilizing animal models. This Special Issue of *Veterinary Sciences* on “Allergies in Animals and Humans” invited articles and reviews, especially on how allergic diseases in animals can shed light on these in humans, or visa versa. 

The review by Marsella and De Benedetto [1] gives an up to date summary on the similarities and differences of atopic dermatitis between dogs, cats, horses, and humans in terms of pathological mechanisms and current and new treatments. The authors quite rightly point out that canine atopic dermatitis, although associated with characteristic clinical signs and allergic sensitization to a number of inhalant or food allergens, is not a single disease entity but is a clinical syndrome characterized by erythema and pruritus caused by a multitude of different factors. Thus treatment and management requires a multimodal approach which Marsella and De Benedetto comprehensively discuss [1]. Intriguingly, feline atopic dermatitis manifests in different ways compared to other animal and human species and the skin barrier defect documented in dogs, horses, and humans has not been documented in cats. A number of other differences of atopic dermatitis characteristics between the four species have also been documented, such as associations of atopic dermatitis with flea allergy in dogs and cats and progression of the atopic march in horses and humans. Thus, there are a number of similarities, but also differences, in pathological mechanisms of atopic dermatitis between animal species and humans that may increase our understanding of this complex syndrome.

Specific IgE sensitization to animal allergens is a known risk factor for the development of asthma and rhino-conjunctivitis in humans. As Zahradnik and Raulf [2] point out more than half of the worldwide population keep at least one pet, such as dogs and cats, at home. Additionally, exposure to animal allergens can also occur on farms, such as to horses and cows; and at research institutes, such as to mice and rats. In their review of respiratory allergens from furred mammals, Zahradnik and Raulf first overview these allergens according to protein families. They then comprehensively review the published studies on the presence of animal allergens in such diverse places such as laboratory animal facilities, veterinary clinics, farms, domestic dwelling, and public buildings and transport. Thus, exposure to animal allergens can occur in a wide range of environments and are subject to various environmental factors as well as demonstrating a high variability. Knowledge of these factors could help in allergen avoidance by those sensitized and symptomatic to animal allergens.

As already mentioned, feline atopic dermatitis differs from atopic dermatitis in dogs and humans in a number of mechanisms and presentation, which poses a challenge to veterinarians. In her review, Diesel [3] presents what is known regarding allergic skin disease in cats, focussing predominantly on atopic dermatitis. As the author points out flea and food-induced hypersensitivity dermatitis is similar between dogs and cats. However, feline atopic dermatitis, although sharing some similarities with canine and human atopic dermatitis, differs in regard to pathogenesis, clinical manifestations, and treatment.

The incidence of drug hypersensitivity reactions in small animals is suggested to range from 0.1% to 3%, similar to what has been described in humans. In patients with suspected drug hypersensitivity, a diagnosis is established by a careful medical examination and a detailed medical and pharmacological history. A number of in vivo tests have been developed in human drug hypersensitivity that can help confirm the diagnosis and may identify the culprit drug. Lavergne [4] critically discusses these in vivo tests in regard to strengths and weaknesses, specificity and sensitivity, and practical aspects and limitations, for potential use in the small animal veterinary clinic. The author also points out that type 1 drug hypersensitivity in veterinary medicine is a neglected field and that these in vivo tests, as validated for human patients, will have to be scientifically validated in veterinary patients.

Insect bite hypersensitivity is a common allergic reaction affecting horses and ponies impairing their quality of life. In their article, Lomas and Robinson [5], using qualitative research methodology, conducted a pilot study exploring the impact of equine insect bite hypersensitivity from the perspective of various stakeholders in the equine industry. Although limited by the small number of stakeholder participants (nine) and the small geographical area they came from, this study did produce some interesting findings. The majority of respondents lacked specific knowledge regarding the causative factor for equine insect bite hypersensitivity (hypersensitivity reaction to the saliva of the *Culicoides* biting midge). Specific knowledge of equine insect bite hypersensitivity aetiology should result in better prevention and management of this condition.

A closer collaboration between researchers in the veterinary and medical sciences, may thus lead to renewed ideas to tackle these important disorders.
